# The tumour microenvironment influences long-term tamoxifen benefit in postmenopausal ER+/HER2− breast cancer patients: a secondary analysis of the randomised Stockholm Tamoxifen (STO-3) trial

**DOI:** 10.1186/s12916-026-05091-5

**Published:** 2026-08-05

**Authors:** Paula Camargo-Romera, Miguel Castresana‑Aguirre, Oscar Danielsson, Huma Dar, Arne Östman, Kamila Czene, Linda S. Lindström, Nicholas P. Tobin

**Affiliations:** 1 Department of Oncology and Pathology, BioClinicum, Karolinska Institutet and University Hospital, Visionsgatan 4, 171 64 Stockholm, Sweden; 2 Breast Center, Karolinska Comprehensive Cancer Center, Karolinska University Hospital, Stockholm, Sweden; 3 Department of Medical Epidemiology and Biostatistics, Karolinska Institutet, Stockholm, Sweden

**Keywords:** TME, Breast cancer, Tamoxifen benefit, Endocrine response, Deconvolution algorithms

## Abstract

**Background:**

The tumour microenvironment (TME) influences breast cancer progression and treatment response. We investigated whether TME composition predicts tamoxifen benefit in postmenopausal women with oestrogen receptor-positive, HER2-negative (ER+HER2-) breast cancer.

**Methods:**

This study included 513 patients from the Stockholm Tamoxifen (STO-3) trial, which randomised postmenopausal, lymph node–negative women to tamoxifen or no endocrine therapy. Bulk tumour transcriptomes were deconvoluted with the ConsensusTME algorithm to estimate the relative abundance of 18 immune and stromal cell types. A summary score of combined immune cells was created on a per patient basis and evaluated alongside fibroblast and endothelial stromal compartments. Patients were categorised into immune and stromal tertiles on the basis of these scores. Associations between TME composition and tumour characteristics were evaluated using Spearman correlations and Fisher’s exact test. Tamoxifen benefit was analysed by univariable Kaplan-Meier (log-rank) and multivariable Cox proportional hazards adjusting for age, tumour size, grade, progesterone receptor, Ki-67, and radiotherapy. Differential expression was assessed with limma and pathway enrichment with fgsea using Hallmark gene sets from MSigDB.

**Results:**

Low immune abundance was significantly associated with higher ER expression (Kruskal-Wallis test p < 0.001). Among tamoxifen-treated patients, those with low immune scores showed improved distant recurrence-free interval (DRFI) relative to untreated patients (log-rank p < 0.001). Similarly, intermediate endothelial (p < 0.001) and low/intermediate fibroblast abundances (p = 0.042, p = 0.009) were associated with favourable DRFI. In multivariable models, low immune (adjusted HR = 0.17, 95% CI 0.08–0.40), intermediate endothelial (aHR = 0.21, 95% CI 0.09–0.51), and low/intermediate fibroblast tertiles (aHR = 0.50, 95% CI 0.27–0.93; aHR = 0.36, 95% CI 0.17–0.77) retained significance. Transcriptomic analysis revealed enrichment of oestrogen-response, MYC-target, and oxidative-phosphorylation pathways in low-immune and low-fibroblast tumours, while interferon-γ response and allograft rejection pathways were downregulated.

**Conclusions:**

TME composition modulates tamoxifen benefit in postmenopausal ER+HER2− breast cancer. Low immune, intermediate endothelial, and low/intermediate fibroblast abundances are associated with improved benefit from tamoxifen, suggesting that both immune and stromal compartments influence endocrine treatment efficacy.

## Background

Breast cancer is one of the most common types of female cancers and is a principal cause of mortality in women globally [1, 2]. Understanding the biology of breast cancer poses a challenge owing to its heterogeneous nature, where individual tumours exhibit diverse characteristics and behaviours [3]. This complexity is further compounded by the influence of the tumour microenvironment (TME), making it challenging to unravel disease mechanisms and develop effective treatment strategies [3].

The TME is comprised of tumour, immune and stromal cells along with the extracellular matrix (a non-cellular component) [4]. It plays a crucial role in breast cancer proliferation, immune system suppression, response to treatment [5] and disease progression [6]. Importantly, the cellular composition and abundance of TME cells have been shown to vary depending on tumour oestrogen receptor status [7] and this variability has been shown to influence the survival of breast cancer patients. In ER-positive tumours with up to 10 years of median follow-up, patients with a higher abundance of M0 macrophages show poorer survival than those with lower abundances [8]. Conversely, in ER-negative tumours, the presence of CD8+ and activated memory T cells have been associated with better survival outcomes [8–11]. How these subtypes influence the long term (>25 year) survival of breast cancer patients is currently unclear.

These studies highlight the importance of examining the TME separately on the basis of ER status. To date however, studies relating TME cell abundance to treatment response in randomised clinical trial samples from ER-positive tumours are lacking. Here, we perform a comprehensive analysis of how tumour microenvironment (TME) composition influences the benefit derived from tamoxifen treatment in ER-positive, HER2-negative postmenopausal breast cancer patients within the randomised STO-3 trial. This trial uniquely includes a no-endocrine-therapy control arm enabling direct comparison of treatment benefit. We apply a deconvolution technique to determine the relative abundance of eighteen TME cell types and perform uni- and multi-variable survival analyses to determine their impact on tamoxifen-treated patient survival relative to non-tamoxifen treated patients.

## Methods

### The Stockholm Tamoxifen clinical trial

**Fig. 1:**
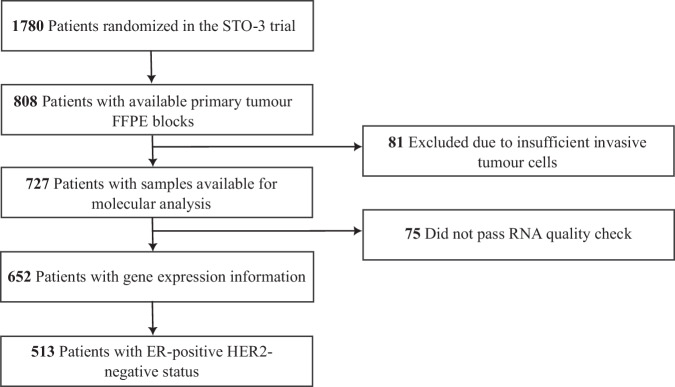
CONSORT diagram of the Stockholm tamoxifen randomised trial (STO-3 trial). ER, oestrogen receptor. HER2, human epidermal growth factor receptor 2

The Stockholm Breast Cancer Study Group conducted randomised trials from 1976 and onwards [12, 13]. The Stockholm Tamoxifen Trial 3 (STO-3) enrolled 1780 postmenopausal patients with lymph node-negative breast cancer who were randomised to two years of adjuvant tamoxifen (40 mg daily) or no adjuvant treatment [12, 13]. In 1983, patients who re-consented and were disease-free after 2 years of tamoxifen treatment were randomised to 3 more years of tamoxifen or no further treatment [12, 13]. A subset of 808 patients with formalin-fixed paraffin-embedded (FFPE) material was available for molecular analyses, and this patient subset is well-balanced relative to the original STO-3 trial regarding tumour characteristics [13, 14]. Samples from eighty-one of these patients were excluded due to insufficient invasive tumour cells, leaving 727 samples in total [14] (Fig. 1). Comprehensive patient and clinicopathological annotations were present for all participants [13]. Follow-up until December 31, 2016, was obtained from Swedish national and regional registries of high validity and essentially complete coverage [15–17]. Detailed patient and clinical information was available for all patients in the trials. The STO-trials were approved by the Karolinska Institutet Regional Ethics Committee with the Stockholm Regional Cancer Center as the trial center. Informed consent was obtained before randomization. The trials were approved and initiated before the practice of trial registration in Sweden [15–17].

### Immunohistochemistry

Immunohistochemical analysis for ER, PR, HER2, and Ki-67 was performed in 2014 (STO-3) [13]. The percentage of cancer cells positive for ER, PR, HER2, and Ki-67 was scored by experienced breast cancer pathologists. ER-positive and PR-positive status were defined by a threshold of 10% or greater according to Swedish National guidelines [18], and Ki-67 was measured in the whole tumour section and categorised as low (<15%) and high (≥15%). HER2 positivity was defined as intensity 3+ by IHC.

### Tumour size and tumour grade

Tumour diameter was measured according to clinical guidelines and classified into three groups: T1a/b (≤10 mm), T1c (11–20 mm), and T2-T3 (>20 mm). Tumour grade was assessed according to the system (Elston grade) [19].

### Agilent microarray gene expression profiling and 70-gene signature generation

Agilent microarray profiling was performed on FFPE breast cancer tumour tissue in 2014 [13] using custom-designed arrays from Agilent Technologies (Santa Clara, CA). Each array contained ~32.1 K probes representing ~21.5 K unique genes. Of the 727 breast cancer tumours examined, 652 successfully passed the RNA quality check according to the diagnostic quality model. This filtering process was utilised to ensure that the transcriptomic data reflected reliable biological signals and to mitigate potential artefacts arising from the archival age of the FFPE blocks. Out of these, 513 tumours were ER-positive/HER2-negative (Fig. 1). A continuous 70-gene signature score was derived from this microarray data using the genefu R package and the resulting variable was scaled between 0 and 1 for reporting in relation to TME subtype tertiles (Table 2) and exploratory multivariable analyses.

### Deconvolution of tumour microenvironment and immune score construction

The deconvolution algorithm Consensus^TME^ [20] was employed to discern the composition of TME cell types from bulk microarray data and was chosen owing to its cancer specificity and integration of multiple methodologies. The algorithm outputs normalized enrichment score (NES) scores on a per tumour basis that reflects the relative abundance of 18 TME cell types. These include Regulatory T cells, γδ T cells, B cells, CD4+T cells, CD8+T cells, Cytotoxic cells, Dendritic cells, Eosinophils, Macrophages, M1 macrophages, M2 macrophages, Mast cells, Monocytes, Neutrophils, NK cells, Plasma cells, Fibroblasts, and Endothelial cells.

As Consensus^TME^ estimates are comparable across patients but not between cell types, we first applied z-score normalisation to each cell type across all patients. This ensured that each immune lineage contributed equally to the aggregated immune score, avoiding dominance by cell types with larger raw numerical ranges. The resulting standardised scores were then summed across all immune cell types (e.g. all cell types noted above with the exception of fibroblasts, and endothelial cells) within each tumour sample to construct an aggregated immune score. This continuous variable was subsequently split into tertiles of low, intermediate and high relative immune abundance and used for survival and differential gene expression analyses. The same tertile split was applied to the continuous NES scores for fibroblast and endothelial cell abundances. Finally, a semi-supervised hierarchical clustering was performed using the average agglomeration method (UPGMA) to indicate similarity in immune abundance between samples clustering columns on the basis of immune score tertiles and rows on the basis of Pearson correlation.

### Differential expression and gene set enrichment analysis

To examine genes and pathways associated with distinct tumour microenvironment cell type compositions, we performed differential gene expression and pathway enrichment analyses based on the immune, endothelial, and fibroblast tertiles. For the immune and fibroblast scores, samples exhibiting low vs high relative cell-type abundance were compared to capture genes differentially expressed between these extremes. For the endothelial score, the intermediate group was compared to low and high separately, in line with results from survival analyses (see Results). Differential expression analysis was performed using the limma package (version 3.62.2) in R, employing empirical Bayes moderation to estimate log2 fold changes and associated statistical significance. Genes were subsequently ranked by their moderated t-statistics. To ensure that differentially expressed genes did not substantially overlap with genes used to define Consensus^TME^ cell-type signatures, we calculated the Jaccard similarity index between these sets. Pathway enrichment analysis was carried out using the fgsea package (version 1.32.4), with curated gene sets sourced from the MSigDB database via the msigdbr package (version 10.0.1). Specifically, the Hallmark gene sets (collection ‘H’) were utilized to identify key biological pathways enriched among the differentially expressed genes. Enrichment significance was assessed using the Benjamini–Hochberg method to control the false discovery rate (FDR).

### Statistical analysis

To test for correlations between NES scores and TME abundances, we performed a Spearman correlation analysis, clustering based on the average agglomeration method (UPGMA), with p < 0.05 and a two-sided alternative hypothesis. Fisher’s exact test was used to assess differences in patient and tumour characteristics between Immune, Endothelial and Fibroblast tertiles. Differences in distant recurrence between tamoxifen treated vs. non-treated were examined within immune, endothelial and fibroblast tertiles by univariable Kaplan-Meier (log-rank test) and multivariable Cox proportional hazard regression (Wald test) analyses. These analyses were designed to estimate tamoxifen benefit within each TME tertile, rather than overall prognostic effects, by directly comparing treated versus untreated patients. The clinical endpoint of interest for all analyses was distant recurrence-free interval (DRFI), with distant disease, i.e. distant metastatic disease, as the event [21]. Follow-up started at the date of primary breast cancer diagnosis and ended at the date of distant recurrence, date of death, or end of study follow-up (December 31, 2016). Multivariable analyses were adjusted for patient and tumour characteristics, including age, tumour size, tumour grade, progesterone receptor status, Ki-67 status, and the administration of radiotherapy. To evaluate the robustness of our findings, sensitivity analyses were also performed by re-fitting multivariable models on a reduced cohort (excluding n = 58 patients with uncertain ER status) and by additionally adjusting for discrete ER expression percentage (ER%) and scaled 70-gene continuous score. Interaction between the different scores and tamoxifen was tested by including a product term in the Cox proportional hazard regression model. All data preparation and analyses were performed using R version 4.4.2. Kaplan-Meier and Cox proportional hazard analyses were performed using the R survival package, the R survminer package was used for survival curves, and the pheatmap package was used for hierarchical clustering analyses. All statistical tests were two-sided; statistical significance was assessed at α = 0.05.

## Results

### Characterisation of the tumour microenvironment

A total of 513 ER+HER2− postmenopausal women from the STO-3 trial with associated microarray-based transcriptomic data were included in this study (Fig. 1). No significant differences in patient and tumour characteristics were observed between those who received tamoxifen treatment and those who did not (Table 1).

**Table 1:** Patient and Tumour Characteristics for 513 Oestrogen Receptor-Positive and HER2 Receptor-Negative Patients in STO-3

		Tamoxifen Untreated (n = 239)	Tamoxifen Treated (n = 274)	p^a^
Age, N (%)	45–54	25 (10.5)	21 (7.7)	0.552
	55–64	118 (49.4)	139 (50.7)	
	65–74	96 (40.1)	114 (41.6)	
Tumour size, N (%)	<=20 mm	191 (79.9)	226 (82.5)	0.746
	>20 mm	45 (18.8)	45 (16.4)	
	Unknown	3 (1.3)	3 (1.1)	
Tumour grade, N (%)	1	59 (24.7)	56 (20.4)	0.609
	2	146 (61.1)	180 (65.7)	
	3	31 (13.0)	33 (12.1)	
	Unknown	3 (1.2)	5 (1.8)	
PR status, N (%)	Positive	170 (71.1)	191 (69.7)	0.928
	Negative	66 (27.6)	80 (29.2)	
	Unknown	3 (1.3)	3 (1.1)	
Ki-67 status, N (%)	Low	55 (23.1)	50 (18.3)	0.209
	High	176 (73.6)	208 (75.9)	
	Unknown	8 (3.3)	16 (5.8)	
Radiotherapy, N (%)	Yes	55 (23.0)	63 (23.0)	1.000
	No	184 (77.0)	211 (77.0)	

^a^p-value calculated by Fisher’s exact test

**Fig. 2:**
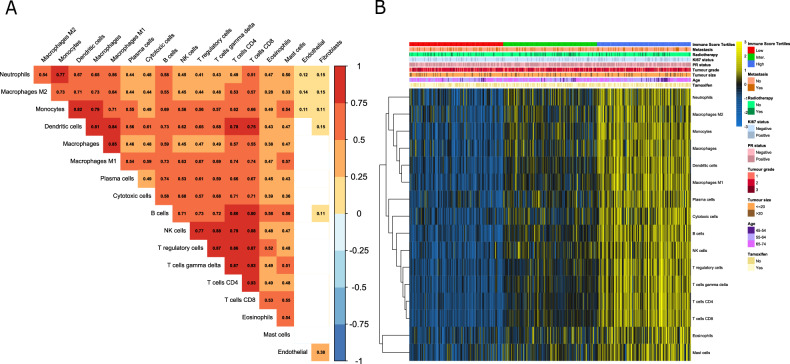
**A** Spearman correlation plot of 18 TME cell types, with coefficients ranging from −1 (negative) to 1 (positive); empty cells indicate non-significant correlations. **B** Semi-supervised heatmap of immune cell types across patient samples, ordered by immune score tertiles. The colour gradient (blue to yellow) reflects relative abundance. Top annotations show clinical and molecular features: metastasis, radiotherapy, Ki-67, PR status, grade, age, and tamoxifen treatment

The Consensus^TME^ deconvolution was applied to the bulk tumour microarray data and yielded normalized enrichment scores (NES) for 18 immune and stromal cell types of the tumour microenvironment on a per patient basis. In order to understand how these cell types relate to each other, we first performed a correlation analysis across all samples. We found that whilst in general immune cell types were significantly and intermediate to strongly correlated with each other (Spearman correlation >0.5 for most comparisons, p < 0.05), no strong correlations were found between stromal cell types (endothelial and fibroblasts) and immune cell types (Fig. 2A, Spearman correlation <0.4 for all stromal vs. immune comparisons). Similarly, an unsupervised clustering of all samples showed immune cell types grouping separately from stromal cell types (Additional file 1: Fig. S1, see dendrogram on the left-hand side, red arrows indicate stromal cell types). Based on these results, we chose to analyse immune cell types together and separately from their stromal counterparts.

Given the high correlation between immune cell types, we next constructed an aggregated immune score by summing the values for all immune cell types and dividing the resulting continuous variable into tertiles of low, intermediate and high immune expression. A semi-supervised heatmap of immune cell types ordered by tertiles is shown in Fig. 2B where again the similarities in immune cell expression within each tertile are readily apparent (Fig. 2B, tertile proportions shown highest up on column legend). Tertiles were similarly constructed for endothelial and fibroblast continuous variables separately. When examining tumour characteristics in relation to these tertile groups, we found a significantly higher percentage of ER-positive cells and continuous 70-gene signature score in the immune score low tertile (Kruskal-Wallis test, p < 0.001 and p = 0.0185, respectively Table 2). Furthermore, statistically significant differences were noted between endothelial and fibroblast tertiles and tumour size, grade, Ki-67, clinical risk and 70-gene signature score (Table 2, Fisher’s exact test, p < 0.001 for most comparisons). In general, low levels of both endothelial and fibroblast cell types were associated with more aggressive tumour characteristics (e.g. larger tumour size, grade 3 status and higher clinical risk [22]).

**Table 2:** Tumour characteristics for STO-3 between the tertiles of Immune, Endothelial and Fibroblast scores

		Immune Low (N = 171)	Immune Inter. (N = 171)	Immune High (N = 171)	p^a^	Endothelial Low (N = 171)	Endothelial Inter. (N = 171)	Endothelial High (N = 171)	p^a^	Fibroblast Low (N = 171)	Fibroblast Inter. (N = 171)	Fibroblast High (N = 171)	p^a^
ER %, Mean ± SEM		80.9 ± 1.3	77.4 ± 1.7	73.3 ± 1.7	**<0.001**	77.9 ± 1.5	77.8 ± 1.6	75.9 ± 1.8	0.899	79.3 ± 1.4	74.4 ± 1.9	78.0 ± 1.4	0.252
	Unknown	15	29	22		16	19	31		17	22	27	
PR status, N (%)	Positive	121 (70.8)	121 (70.7)	119 (69.6)	0.953	123 (71.9)	125 (73.1)	113 (66.1)	0.219	119 (69.6)	123 (71.9)	119 (69.6)	0.868
	Negative	49 (28.7)	48 (28.1)	49 (28.6)		48 (28.1)	44 (25.7)	54 (31.6)		51 (29.8)	46 (26.9)	49 (28.7)	
	Unknown	1 (0.5)	2 (1.2)	3 (1.8)		0 (0.0)	2 (1.2)	4 (2.3)		1 (0.6)	2 (1.2)	3 (1.7)	
Tumour size, N (%)	<=20	137 (80.1)	139 (81.3)	141 (82.5)	0.983	124 (72.5)	140 (81.9)	153 (89.5)	**<0.001**	124 (72.5)	143 (83.6)	150 (87.7)	**<0.001**
	>20	32 (18.7)	30 (17.5)	28 (16.3)		47 (27.5)	27 (15.8)	16 (9.4)		45 (26.3)	28 (16.4)	17 (9.9)	
	Unknown	2 (1.2)	2 (1.2)	2 (1.2)		0 (0.0)	4 (2.3)	2 (1.1)		2 (1.2)	0 (0.0)	4 (2.4)	
Tumour grade, N (%)	1	37 (21.6)	42 (24.6)	36 (21.1)	0.944	25 (14.6)	33 (19.3)	57 (33.3)	**<0.001**	13 (7.6)	36 (21.1)	66 (38.6)	**<0.001**
	2	111 (64.9)	104 (60.8)	111 (64.8)		107 (62.6)	114 (66.7)	105 (61.4)		114 (66.7)	112 (65.5)	100 (58.5)	
	3	21 (12.3)	21 (12.3)	22 (12.9)		35 (20.5)	23 (13.5)	6 (3.5)		41 (24.0)	19 (11.1)	4 (2.3)	
	Unknown	2 (1.2)	4 (2.3)	2 (1.2)		4 (2.3)	1 (0.5)	3 (1.8)		3 (1.7)	4 (2.3)	1 (0.6)	
Ki-67 status, N (%)	Low	35 (20.5)	29 (17.0)	41 (24.0)	0.488	47 (27.5)	36 (21.1)	22 (12.9)	**0.006**	57 (33.4)	32 (18.7)	16 (9.4)	**<0.001**
	High	130 (76.0)	133 (77.7)	121 (70.7)		120 (70.2)	127 (74.3)	137 (80.1)		109 (63.7)	130 (76.0)	145 (84.8)	
	Unknown	6 (3.5)	9 (5.3)	9 (5.3)		4 (2.3)	8 (4.6)	12 (7.0)		5 (2.9)	9 (5.3)	10 (5.8)	
MINDACT clinical risk, N (%)	C-high	36 (21.1)	36 (21.1)	40 (23.4)	0.936	58 (33.9)	38 (22.2)	16 (9.4)	**<0.001**	62 (36.3)	32 (18.7)	18 (10.5)	**<0.001**
	C-low	131 (76.6)	130 (76.0)	128 (74.8)		109 (63.7)	128 (74.9)	152 (88.8)		105 (61.4)	135 (78.9)	149 (87.1)	
	Unknown	4 (2.3)	5 (2.9)	3 (1.8)		4 (2.4)	5 (2.9)	3 (1.8)		4 (2.3)	4 (2.4)	4 (2.4)	
70-gene sig., Mean ± SEM		0.61 ± 0.01	0.60 ± 0.01	0.56 ± 0.01	**0.0185**	0.55 ± 0.01	0.59 ± 0.01	0.63 ± 0.01	**<0.001**	0.51 ± 0.01	0.60 ± 0.01	0.66 ± 0.01	**<0.001**

^a^p-value calculated by Fisher’s exact test for all variables except for the ER %, PR % and 70-gene sig. Instead, Kruskal-Wallis was used

### Long-term tamoxifen benefit within TME tertiles

**Fig. 3:**
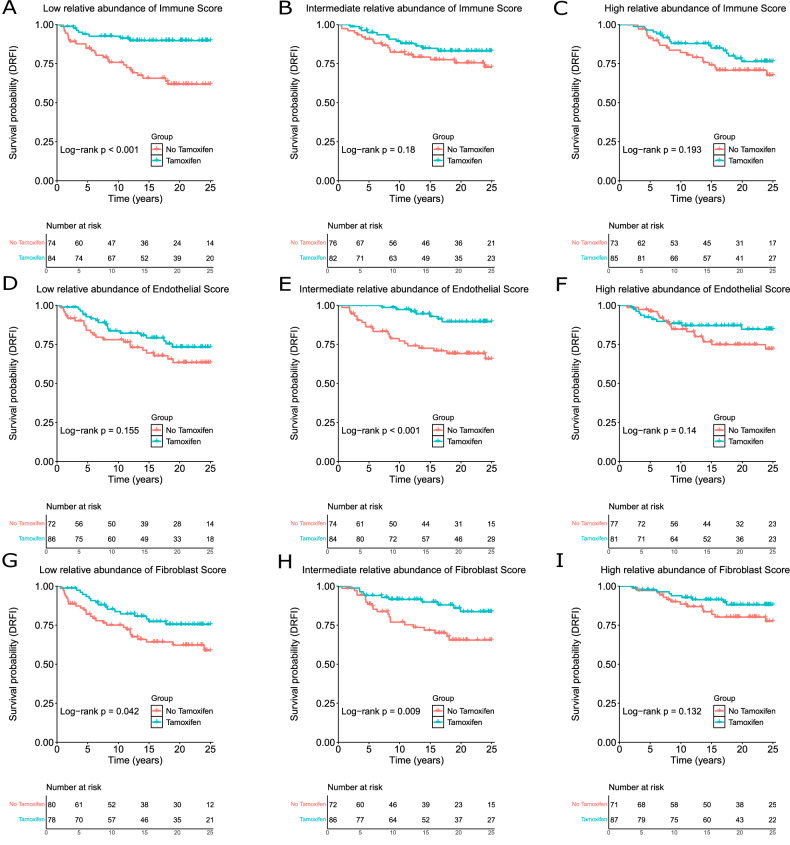
Univariable Kaplan-Meier analysis of DRFI in tamoxifen-treated patients compared to non-tamoxifen treated patients across tertile scores. Comparisons represent treatment benefit within each TME tertile (tamoxifen-treated vs. untreated). **A**-**C** Low, Intermediate and High relative abundance tertiles for the Immune score. **D**-**F** Low, Intermediate and High relative abundance tertiles for the Endothelial score. **G**-**I** Low, Intermediate and High relative abundance tertiles for the Fibroblast score

Next, we assessed tamoxifen benefit - defined as differences in distant recurrence-free interval (DRFI) between treated and untreated patients - across immune, endothelial, and fibroblast tertiles, as well as within individual immune cell type tertiles. Univariable Kaplan–Meier analyses demonstrated a statistically significant improvement in long-term DRFI in/among tamoxifen-treated patients with lower relative immune score abundances but not intermediate or high, compared to patients who did not receive tamoxifen therapy (log-rank p < 0.001, for tamoxifen immune score low tertile vs. untreated patients, Fig. 3A-C). Similarly, tamoxifen conferred a significant benefit in the endothelial intermediate tertile (log-rank p < 0.001, Fig. 3E) and fibroblast low and intermediate tertiles (log-rank p = 0.042 and p = 0.009, respectively, Fig. 3G-H) relative to patients who did not receive tamoxifen therapy. Kaplan–Meier analyses of distant recurrence by individual immune cell types revealed a similar trend to the aggregated immune score, whereby a statistically significant tamoxifen benefit was observed in the majority of immune cell low but not high tertiles (log-rank p < 0.05 for all comparisons, Additional file 2: Fig. S2A-P, adjusted for multiple testing using Bonferroni correction).

**Fig. 4:**
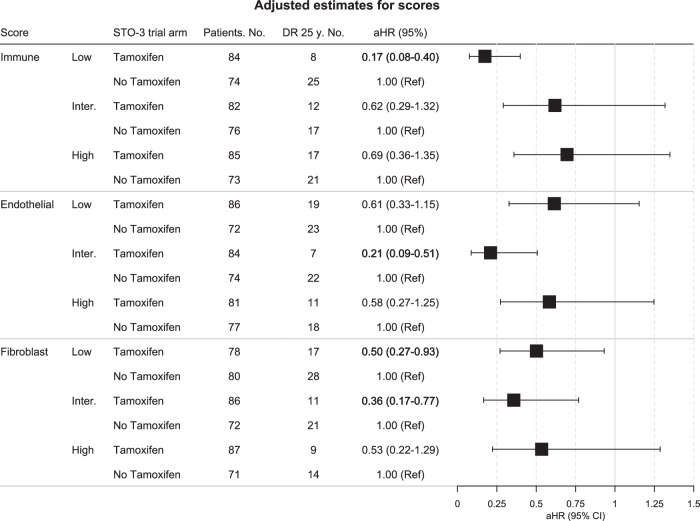
Multivariable Cox proportional hazard regression analysis of DRFI in tamoxifen-treated patients compared to untreated patients across tertile scores. Multivariable analyses were adjusted for clinical information and tumour characteristics, including age, tumour size, tumour grade, progesterone receptor status, and Ki-67 status, and the administration of radiotherapy. DRFI, distant recurrence-free interval; DR, distant recurrence; aHR, adjusted hazard ratio; Ref, reference

Similar findings were seen in multivariable Cox proportional hazard modelling where tamoxifen conferred significantly improved long-term DRFI in tumours with lower immune cell abundance (adjusted Hazard Ratio (aHR) = 0.17; 95% CI, 0.08–0.40), intermediate endothelial cell abundance (aHR = 0.21; 95% CI, 0.09–0.51), and both low and intermediate fibroblast abundances (aHR = 0.50; 95% CI, 0.27–0.93, and aHR = 0.36; 95% CI, 0.17–0.77, respectively), compared to untreated patients (Fig. 4). Similarly, individual immune cell types also revealed improved DRFI for tamoxifen-treated patients for all immune cell low tertiles (Additional file 3: Fig. S3), with some of the strongest associations observed for patients with low relative abundances of M1 macrophages (aHR = 0.13; 95% CI, 0.05–0.35), mast cells (aHR = 0.16; 95% CI, 0.06–0.43), and eosinophils (aHR = 0.14; 95% CI, 0.05–0.36). A significant interaction between immune tertiles and tamoxifen treatment was also observed (p = 0.008), indicating that the impact of immune infiltration differs between treated and untreated patients and that tamoxifen benefit is greatest in the immune-low tertile. By contrast, no statistically significant interaction was found between tamoxifen treatment and endothelial or fibroblast tertiles (p = 0.850 and p = 0.800, respectively), suggesting predominantly prognostic rather than treatment-modifying effects for these scores. Finally, additionally adjusting for the continuous 70-gene signature did not alter these conclusions (data not shown) and sensitivity analyses in a reduced cohort for which ER percentage was available showed that the associations for immune and endothelial scores remained stable after adjusting for this variable (Additional file 4: Table S1). Although the fibroblast-low subgroup lost statistical significance in this reduced cohort, this was likely due to decreased statistical power rather than a true weakening of the association.

### Distinct molecular profiles associated with tumour composition

**Fig. 5:**
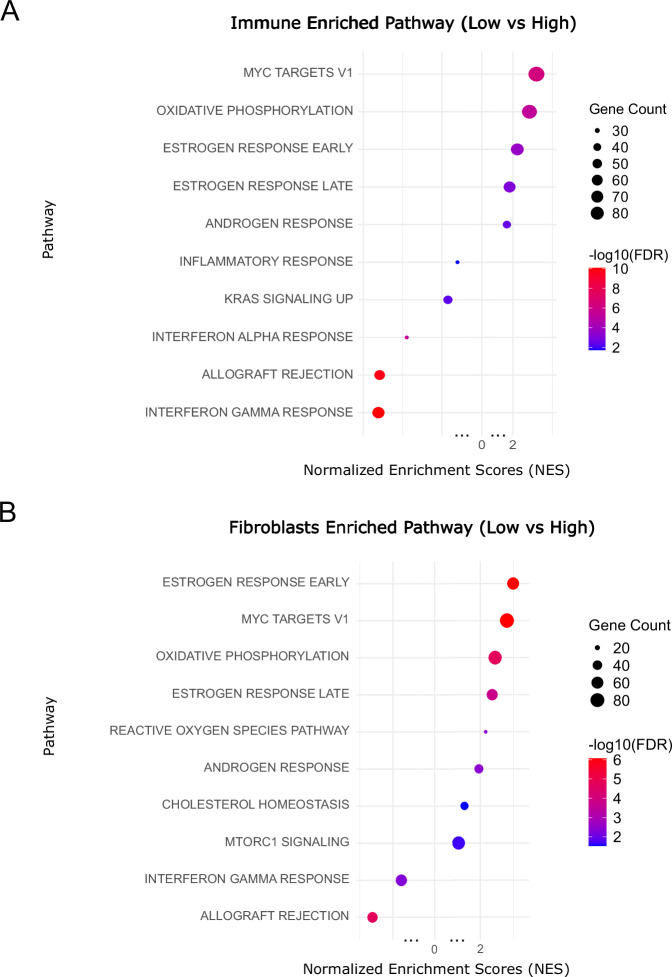
Dot plot representation of the most and least enriched pathways in low vs high for **A** immune score, and **B** fibroblast score. The circle’s size represents the number of genes in each pathway, and the colour of the qscore for each pathway

In order to better understand why tamoxifen benefit is observed in specific TME tertiles we next performed differential expression with subsequent pathway enrichment analyses. When comparing low vs. high tertiles for immune and fibroblast scores we found that tumours with low scores for both cell types were enriched for early and late oestrogen response, MYC targets, and oxidative phosphorylation hallmark pathways, while interferon gamma response and allograft rejection were downregulated (Fig. 5). Endothelial intermediate tumours were compared to low and high subgroups separately and MYC targets along with the oxidative phosphorylation were the only statistically significant downregulated pathways in both analyses (Additional file 5: Fig. S4). Interestingly, hallmark oestrogen response early and late pathways were downregulated in the intermediate compared to low tertile for endothelial scores, although this difference was not significant when comparing intermediate to high. Finally, to ensure that differentially expressed genes were not the same as those that define the ConsensusTME cell type signatures we applied the Jaccard similarity index. No significant overlap was found between the immune-, fibroblast-, and endothelial-associated genes and the differentially expressed genes underlying their respective enriched pathways, suggesting distinct molecular processes for each pathway enrichment (data not shown).

## Discussion

In this study, we examined the long-term benefit of tamoxifen treatment according to tumour microenvironment (TME) baseline composition in postmenopausal patients with ER+HER2− breast cancer. Using the randomised design of the STO-3 trial including a control arm with no endocrine therapy, we compared tamoxifen-treated patients versus untreated across tertiles of immune, endothelial, and fibroblast abundance. We found that low immune cell, intermediate endothelial cell, and low to intermediate fibroblast cell abundances are each associated with a greater long-term benefit from tamoxifen. Moreover, we identified differentially expressed hallmark pathways in these groups, including the enrichment of oestrogen response, MYC targets and oxidative phosphorylation in immune and fibroblast low tertiles.

Comparisons with previous studies are challenging due to the unique long-term follow-up of our cohort and the direct clinical trial arm comparisons within immune and stromal abundance tertiles. Nevertheless, several studies align with our finding that low immune cell abundance may be linked to greater tamoxifen benefit in ER-positive breast cancer. Ali et al. used CIBERSORT to show that ER-positive tumours lacking immune infiltration had distinct biology and intermediate survival outcomes [8]. They reported poorer survival with increased M0 macrophages and regulatory T cells on the ER+HER- group [8]. Similarly, Sobral-Leite et al. observed that a higher abundance of CD4+ or CD8+T-cells were associated with poorer prognosis in ER+HER2− low grade breast cancer patients from a randomised clinical trial [23]. Heindl et al. also found that high immune spatial scores (but not abundance scores) were associated with worse 10-year survival in the ATAC cohort [24]. Collectively, these studies support our conclusion that immunologically low ER-positive HER2-negative tumours may have distinct biology associated with greater tamoxifen benefit.

Our work also identified a number of pathways enriched in tumours with low immune and fibroblast abundance (vs. high) and intermediate endothelial abundance (vs. low and high), some of which align with known biology for tamoxifen response. We found higher levels of oestrogen response pathways (in addition to a higher percentage of oestrogen positive cells) in tumours with a lower immune and fibroblast scores. This is likely directly related to the DRFI improvement found following tamoxifen treatment for these patient subgroups (Figs. 3 and 4) and is consistent with studies showing that tumours with a higher percentage of ER-positive cells tend to respond better to endocrine therapy [25, 26]. Similarly, the higher levels of oxidative phosphorylation and lower levels of interferon gamma pathways in these tumours are in line with previous associations to improved efficacy of tamoxifen [27, 28]. Notably, the significant interaction between immune tertiles and tamoxifen treatment is consistent with a predictive component to these associations, indicating that the effect of immune infiltration on outcome differs according to treatment group. Contrastingly however, oxidative phosphorylation was depleted in tumours with intermediate endothelial cell abundance. Of note, only ~25% of endothelial intermediate tumours were also immune low which may help to explain these opposing findings (data not shown). Interpretation of intermediate biomarker expression is difficult however and we would urge caution in over-interpreting this specific result until independently validated. A more general clinical interpretation of our findings could be that low immune and fibroblast content may reflect reduced stromal remodeling and fewer physical or signaling barriers to therapy [29]. Taken together, these observations suggest that the magnitude of tamoxifen benefit differs across TME groups. Tumours with low immune infiltration, which are at higher baseline risk, appear to derive greater benefit from tamoxifen, whereas those with higher immune abundance show a more limited incremental benefit.

Our results also have potential implications for treatment stratification in hormone receptor-positive breast cancer. The observation that tumours with low immune infiltration derive greater benefit from tamoxifen, while those with higher immune abundance do not, suggests that baseline TME composition may reflect underlying differences in endocrine sensitivity. Immune-low or immune “cold” tumours may represent a more endocrine-driven phenotype, whereas immune-high or “hot” tumours could be characterised by alternative biological processes that reduce responsiveness to selective oestrogen receptor modulation. In this context, patients with immune-high tumours may benefit from alternative endocrine strategies, such as aromatase inhibitors or selective oestrogen receptor degraders, or from combination approaches incorporating targeted or immune-based therapies. For example, combinations with CDK4/6 inhibitors, which are now standard in advanced hormone receptor-positive disease [30–33], or immune checkpoint blockade [34, 35] in more immune-infiltrated tumours, may represent promising avenues. Further work is needed to determine whether TME composition can guide the selection of endocrine therapy and identify patients who may benefit from combination treatment approaches.

The strengths of our study include the long-term clinical follow-up of 25 years, the randomised clinical trial design allowing direct comparison to a control arm who did not receive tamoxifen, and the comprehensive nature of our analysis examining 18 TME cell types within a single study. The limitations are as follows: *First*, this study is based on a historical clinical trial cohort, and both treatment practices and patient characteristics have evolved since the initiation of STO-3. The trial was conducted prior to the widespread use of aromatase inhibitors in postmenopausal patients, and both the duration (2 years) and dosing of tamoxifen differ from current standards. As a result, the present study cannot determine whether the associations observed between TME composition and treatment benefit extend to contemporary endocrine therapies, including aromatase inhibitors. In addition, the cohort consists exclusively of postmenopausal, lymph node-negative patients with ER-positive, HER2-negative disease, and therefore the findings may not extend to premenopausal patients or those with node-positive disease. The distribution of tumour size and grade within this cohort may also differ from contemporary clinical populations and should be considered when interpreting these results. Finally, while the archival age of the STO-3 samples presents potential challenges for transcriptomic analysis, we applied a diagnostic quality model to exclude degraded samples and used robust signature-based deconvolution methods to ensure that the reported TME abundances reflect genuine biological variation rather than technical artefact. *Second*, although we analysed tumours from 513 patients in total, subgroup tertile analyses resulted in smaller sample sizes (approximately 70–85 patients per arm), which may limit statistical precision. This is particularly relevant for endothelial and fibroblast subgroups, where lower abundance groups were associated with more aggressive tumour characteristics (Table 2) but contained relatively few patients, and these findings should therefore be interpreted with caution. Reassuringly, however, results for individual immune cell types were consistent with those observed using the aggregated immune score (Additional file 2: Fig. S2 and Additional file 3: Fig. S3). *Third*, a general limitation of deconvolution methods is that they show stronger correlation with fluorescence-activated cell sorting (FACS) for major immune cell populations (e.g. CD4+ and CD8+T cells, B cells, NK cells, and macrophages) than for rarer populations such as regulatory T cells or dendritic cells, which may therefore be less reliably estimated [36]. As our immune cell estimates are derived from bulk gene-expression deconvolution rather than direct quantification by immunohistochemistry or flow cytometry, validation using complementary approaches in contemporary cohorts will be important. *Fourth*, and relatedly, our analysis is based on bulk tumour transcriptomic data and therefore does not capture spatial organisation within the tumour microenvironment. While comprehensive immunohistochemical profiling of multiple immune cell types was beyond the scope of the current study, emerging approaches including spatial and single-cell profiling, as well as artificial intelligence (AI)-based analysis of hematoxylin and eosin (H&E) slides, may provide more detailed characterisation of the TME in future studies [37, 38]. *Fifth* and finally, our analyses are based on transcriptional profiling of archival primary tumour material with long-term clinical follow-up and do not include functional in-vitro or in-vivo validation. As such, these findings should be considered hypothesis-generating with respect to underlying mechanisms and provide a rationale for future preclinical studies to investigate the biology of the TME subgroups identified and to test specific therapeutic strategies suggested by our data. In line with these limitations, future studies should assess whether baseline TME composition predicts benefit from extended endocrine therapy and validate these findings in larger, independent cohorts.

## Conclusions

Findings from the STO-3 trial with 25 years of follow-up demonstrate that baseline TME composition, particularly low immune, intermediate endothelial, and low to intermediate fibroblast abundance, is associated with differential benefit from tamoxifen in ER+HER2− postmenopausal breast cancer. As one of the first studies to assess tamoxifen benefit in relation to the tumour microenvironment within a randomised trial including a no-treatment control arm, these results highlight the importance of microenvironmental context in shaping endocrine therapy response in ER+HER2− disease.

## Supplementary information

Additional file 1, **Fig. S1:** Unsupervised heatmap displaying hierarchical clustering of TME cell types across patient samples. The colour gradient represents scaled values, with blue indicating low and yellow high relative abundance. Annotations above the heatmap denote clinical and molecular features, including Metastasis, Radiotherapy, Ki-67 status, PR status, Tumour grade, Age, and Tamoxifen treatment. Rows correspond to immune cell types, and columns to patients

Additional file 2, **Fig. S2:** Univariable Kaplan-Meier analysis of DRFI in tamoxifen-treated patients compared to patients who did not receive tamoxifen across tertile scores. Letters from A to P represent Low, Intermediate and High relative abundance tertiles for a specific cell type of the tumour microenvironment

Additional file 3, **Fig. S3:** Multivariable Cox proportional hazard regression analysis of DRFI in tamoxifen-treated patients compared to patients who did not receive tamoxifen across cell types in the TME. Multivariable analyses were adjusted for clinical information and tumour characteristics, including age, tumour size, tumour grade, progesterone receptor status, and Ki-67 status, and the administration of radiotherapy. DRFI, distant recurrence-free interval; DR, distant recurrence; aHR, adjusted hazard ratio; Ref, reference

Additional file 4, **Table S1**. Sensitivity analyses of TME scores and tamoxifen benefit. Multivariable Cox regression analysis of DRFI across cell abundance scores. aHRs compare tamoxifen treatment vs. no endocrine therapy across three models: Full cohort, Reduced: Excludes patients with missing/uncertain ER status (n = 58), Adjusted: Reduced cohort with additional adjustment for ER percentage (ER%). ER% is grouped according to proportion scores of positive cells: 0, <1, 1–10, 11–33, 34–66, and ≥67%. TME, Tumour Microenvironment; RFS, Recurrence-Free Survival; aHR, Adjusted Hazard Ratio; CI, Confidence Interval; ER, Estrogen Receptor

Additional file 5, **Fig. S4**. Dot plot representation of the most and least enriched pathways for endothelial cells comparing (A) Low vs Intermediate scores, and (B) Intermediate vs High scores. The circle’s size represents the number of genes in each pathway, and the colour of the qscore for each pathway

## Data Availability

The raw RNA microarray data from the STO randomised controlled trial are managed by the Swedish National Data Service (SND) and can be accessed via 10.5878/3vxa-3c28 [39]. These data cannot be shared publicly to protect the privacy of the individuals who participated in the study; therefore, access is controlled and requires formal evaluation by the STO Trialists Group. All code used for the analyses presented in this manuscript is publicly available at https://github.com/pcr08/STO3_25y_TME_Tamoxifen.

## Abbreviations

AI: Artificial Intelligence
ATAC: Arimidex, Tamoxifen, Alone or in Combination
DRFI: Distant Recurrence-Free Interval
ER: Oestrogen Receptor
ESMO: European Society for Medical Oncology
FFPE: Formalin-Fixed Paraffin-Embedded
HR: Hormone receptor
HER2: Human Epidermal growth factor receptor 2
H&E: Hematoxylin and Eosin
IHC: Immunohistochemistry
NES: Normalized Enrichment Scores
NK: Natural Killer
NO: Nitric Oxide
PR: Progesterone receptor
RNA: Ribonucleic acid
SERM: Selective Oestrogen Receptor Modulator
STO-3: Stockholm tamoxifen randomised trial 3
TAMs: Tumour-Associated Macrophages
TILs: Tumour-infiltrating lymphocytes
TME: Tumour Microenvironment
TNBC: Triple Negative Breast Cancer
UPGMA: Unweighted Pair Group Method with Arithmetic Mean
