# Supplementary material for: The tumour microenvironment influences long-term tamoxifen benefit in postmenopausal ER+/HER2− breast cancer patients: a secondary analysis of the randomised Stockholm Tamoxifen (STO-3) trial
Source: BMC Med. 2026 Aug 5;24:424. doi: 10.1186/s12916-026-05091-5 (PMC13445795; doi:10.1186/s12916-026-05091-5)
Supplement: Supplementary file 1 [file 12916_2026_5091_MOESM1_ESM.pdf]

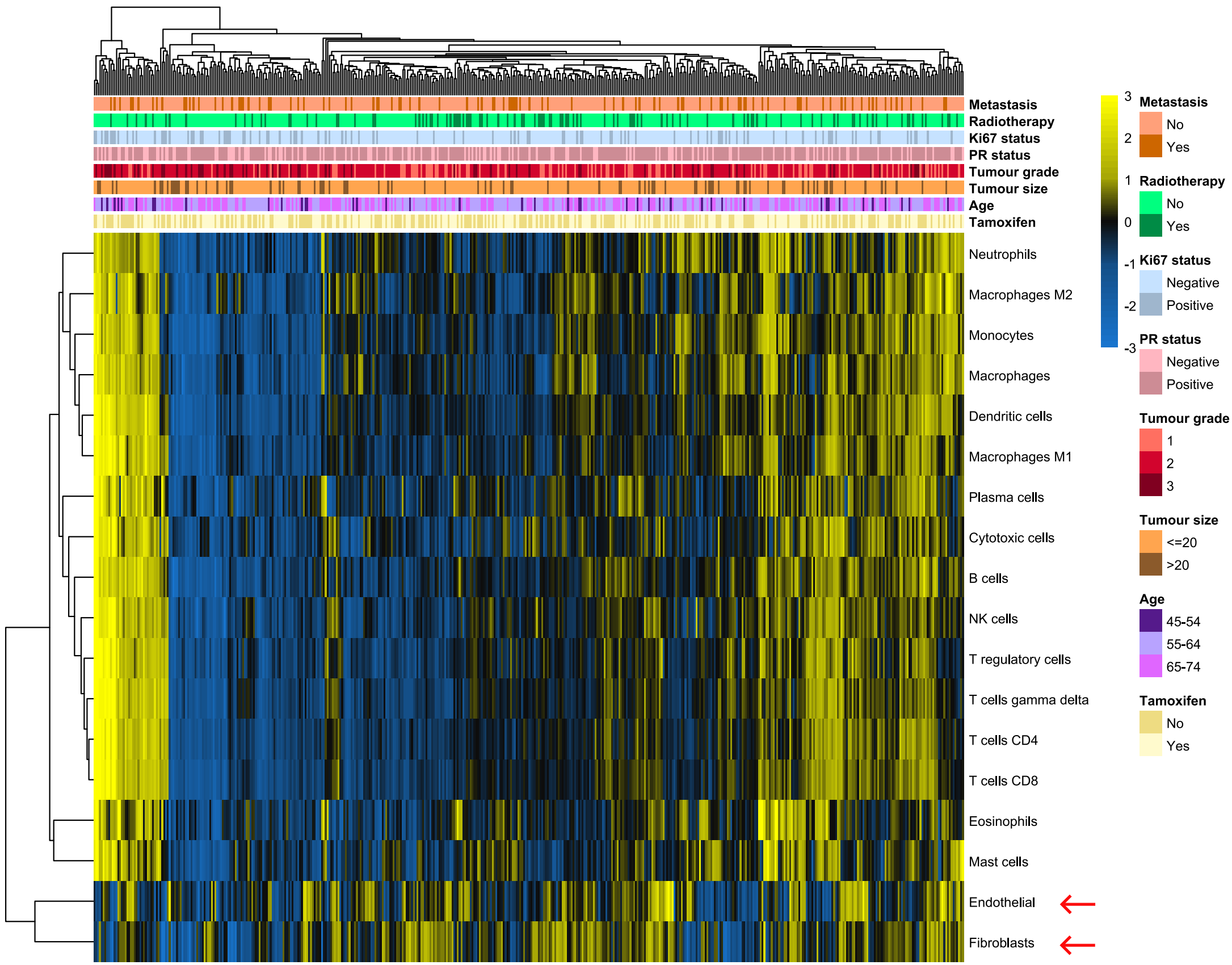

Metastasis
Radiotherapy
Ki67 status
PR status
Tumour grade
Tumour size
Age
Tamoxifen
Neutrophils
Macrophages M2
Monocytes
Macrophages
Dendritic cells
Macrophages M1
Plasma cells
Cytotoxic cells
B cells
NK cells
T regulatory cells
T cells gamma delta
T cells CD4
T cells CD8
Eosinophils
Mast cells
Endothelial
Fibroblasts
3
2
1
0
-1
-2
-3
Metastasis
No
Yes
Radiotherapy
No
Yes
Ki67 status
Negative
Positive
PR status
Negative
Positive
Tumour grade
1
2
3
Tumour size
<=20
>20
Age
45-54
55-64
65-74
Tamoxifen
No
Yes
