# Supplementary material for: The tumour microenvironment influences long-term tamoxifen benefit in postmenopausal ER+/HER2− breast cancer patients: a secondary analysis of the randomised Stockholm Tamoxifen (STO-3) trial
Source: BMC Med. 2026 Aug 5;24:424. doi: 10.1186/s12916-026-05091-5 (PMC13445795; doi:10.1186/s12916-026-05091-5)

Adjusted estimates for cell types

Cell type		STO-3 trial arm	Patients, No.	DR 25 y. No.	aHR (95%)	BH-adj p
B cells	Low	Tamoxifen	86	10	**0.28 (0.13-0.60)**	**0.004**
		No Tamoxifen	72	25	1 (ref.)	
	Inter.	Tamoxifen	80	13	0.45 (0.22-0.91)	0.055
		No Tamoxifen	78	22	1 (ref.)	
	High	Tamoxifen	85	14	0.78 (0.37-1.68)	0.604
		No Tamoxifen	73	16	1 (ref.)	
Cytotoxic cells	Low	Tamoxifen	86	11	**0.30 (0.14-0.63)**	**0.005**
		No Tamoxifen	72	24	1 (ref.)	
	Inter.	Tamoxifen	86	11	**0.32 (0.15-0.70)**	**0.012**
		No Tamoxifen	72	20	1 (ref.)	
	High	Tamoxifen	79	15	0.84 (0.42-1.69)	0.679
		No Tamoxifen	79	19	1 (ref.)	
Dendritic cells	Low	Tamoxifen	90	10	**0.24 (0.11-0.55)**	**0.003**
		No Tamoxifen	68	21	1 (ref.)	
	Inter.	Tamoxifen	78	10	0.46 (0.21-1.01)	0.100
		No Tamoxifen	80	19	1 (ref.)	
	High	Tamoxifen	83	17	0.60 (0.31-1.13)	0.164
		No Tamoxifen	75	23	1 (ref.)	
Eosinophils	Low	Tamoxifen	80	6	**0.14 (0.05-0.36)**	**0.001**
		No Tamoxifen	78	23	1 (ref.)	
	Inter.	Tamoxifen	85	16	0.62 (0.32-1.23)	0.235
		No Tamoxifen	73	20	1 (ref.)	
	High	Tamoxifen	86	15	0.55 (0.27-1.12)	0.154
		No Tamoxifen	72	20	1 (ref.)	
Macrophages	Low	Tamoxifen	86	7	**0.18 (0.08-0.43)**	**0.001**
		No Tamoxifen	72	23	1 (ref.)	
	Inter.	Tamoxifen	79	12	**0.40 (0.20-0.83)**	**0.030**
		No Tamoxifen	79	24	1 (ref.)	
	High	Tamoxifen	86	18	1.09 (0.54-2.22)	0.834
		No Tamoxifen	72	16	1 (ref.)	
Mast cells	Low	Tamoxifen	87	6	**0.16 (0.06-0.43)**	**0.001**
		No Tamoxifen	71	18	1 (ref.)	
	Inter.	Tamoxifen	81	13	0.70 (0.35-1.44)	0.424
		No Tamoxifen	77	20	1 (ref.)	
	High	Tamoxifen	83	18	0.55 (0.30-1.03)	0.107
		No Tamoxifen	75	25	1 (ref.)	
NK cells	Low	Tamoxifen	90	14	**0.39 (0.19-0.80)**	**0.024**
		No Tamoxifen	68	21	1 (ref.)	
	Inter.	Tamoxifen	86	9	0.39 (0.17-0.88)	0.052
		No Tamoxifen	72	20	1 (ref.)	
	High	Tamoxifen	75	14	0.62 (0.30-1.23)	0.235
		No Tamoxifen	83	22	1 (ref.)	
Neutrophils	Low	Tamoxifen	80	7	**0.19 (0.08-0.45)**	**0.001**
		No Tamoxifen	78	25	1 (ref.)	
	Inter.	Tamoxifen	86	12	**0.37 (0.18-0.76)**	**0.018**
		No Tamoxifen	72	21	1 (ref.)	
	High	Tamoxifen	85	18	0.92 (0.45-1.90)	0.841
		No Tamoxifen	73	17	1 (ref.)	
T cells CD4	Low	Tamoxifen	85	9	**0.21 (0.10-0.48)**	**0.001**
		No Tamoxifen	73	24	1 (ref.)	
	Inter.	Tamoxifen	85	12	0.51 (0.25-1.06)	0.122
		No Tamoxifen	73	20	1 (ref.)	
	High	Tamoxifen	81	16	0.74 (0.37-1.48)	0.470
		No Tamoxifen	77	19	1 (ref.)	
T cells CD8	Low	Tamoxifen	92	11	**0.22 (0.10-0.47)**	**0.001**
		No Tamoxifen	66	22	1 (ref.)	
	Inter.	Tamoxifen	81	13	0.52 (0.26-1.03)	0.107
		No Tamoxifen	77	23	1 (ref.)	
	High	Tamoxifen	78	13	0.73 (0.34-1.55)	0.485
		No Tamoxifen	80	18	1 (ref.)	
T cells gamma delta	Low	Tamoxifen	89	9	**0.24 (0.11-0.54)**	**0.003**
		No Tamoxifen	69	21	1 (ref.)	
	Inter.	Tamoxifen	90	14	0.44 (0.22-0.90)	0.052
		No Tamoxifen	68	20	1 (ref.)	
	High	Tamoxifen	72	14	0.74 (0.37-1.46)	0.472
		No Tamoxifen	86	22	1 (ref.)	
T regulatory cells	Low	Tamoxifen	87	9	**0.27 (0.12-0.60)**	**0.005**
		No Tamoxifen	71	21	1 (ref.)	
	Inter.	Tamoxifen	82	14	0.38 (0.19-0.76)	0.018
		No Tamoxifen	76	24	1 (ref.)	
	High	Tamoxifen	82	14	0.79 (0.38-1.66)	0.604
		No Tamoxifen	76	18	1 (ref.)	
Macrophages M1	Low	Tamoxifen	88	6	**0.13 (0.05-0.35)**	**0.001**
		No Tamoxifen	70	23	1 (ref.)	
	Inter.	Tamoxifen	80	16	0.71 (0.37-1.37)	0.398
		No Tamoxifen	78	22	1 (ref.)	
	High	Tamoxifen	83	15	0.66 (0.33-1.33)	0.333
		No Tamoxifen	75	18	1 (ref.)	
Macrophages M2	Low	Tamoxifen	85	9	**0.20 (0.09-0.43)**	**0.001**
		No Tamoxifen	73	27	1 (ref.)	
	Inter.	Tamoxifen	75	13	0.55 (0.27-1.09)	0.139
		No Tamoxifen	83	24	1 (ref.)	
	High	Tamoxifen	91	15	0.93 (0.42-2.06)	0.854
		No Tamoxifen	67	12	1 (ref.)	
Monocytes	Low	Tamoxifen	84	9	**0.19 (0.08-0.45)**	**0.001**
		No Tamoxifen	74	24	1 (ref.)	
	Inter.	Tamoxifen	87	9	**0.28 (0.11-0.72)**	**0.020**
		No Tamoxifen	71	18	1 (ref.)	
	High	Tamoxifen	80	19	0.90 (0.47-1.72)	0.805
		No Tamoxifen	78	21	1 (ref.)	
Plasma cells	Low	Tamoxifen	90	14	**0.23 (0.12-0.47)**	**0.001**
		No Tamoxifen	68	27	1 (ref.)	
	Inter.	Tamoxifen	79	10	0.51 (0.23-1.17)	0.164
		No Tamoxifen	79	16	1 (ref.)	
	High	Tamoxifen	82	13	0.48 (0.23-1.00)	0.097
		No Tamoxifen	76	20	1 (ref.)	

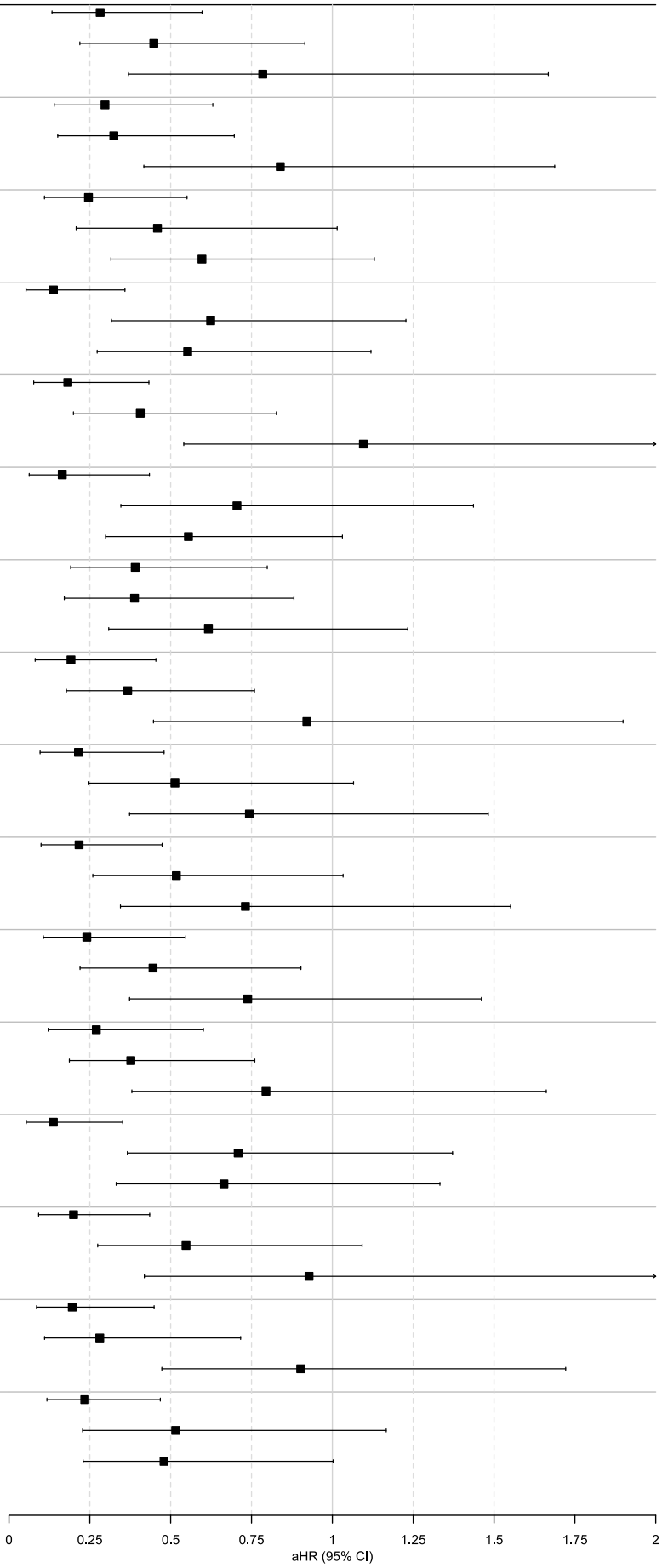

Supplement: Supplementary file 3 [file 12916_2026_5091_MOESM3_ESM.pdf]
