# Supplementary material for: The tumour microenvironment influences long-term tamoxifen benefit in postmenopausal ER+/HER2− breast cancer patients: a secondary analysis of the randomised Stockholm Tamoxifen (STO-3) trial
Source: BMC Med. 2026 Aug 5;24:424. doi: 10.1186/s12916-026-05091-5 (PMC13445795; doi:10.1186/s12916-026-05091-5)
Supplement: Supplementary file 4 [file 12916_2026_5091_MOESM4_ESM.pdf]

Table S1. Sensitivity analyses of TME scores and tamoxifen benefit.

Analysis	Abundance	**Number of patients**	Full cohort aHR (95%)	**Number of patients**	Reduced cohort aHR (95%)	Adjusted cohort aHR (95%)
Immune score	Low	n = 84	**0.17 (0.08-0.40)**	n = 78	**0.14 (0.06-0.35)**	**0.14 (0.05-0.34)**
	Inter.	n = 82	0.62 (0.29-1.32)	n = 70	0.64 (0.29-1.42)	0.67 (0.30-1.50)
	High	n = 85	0.69 (0.36-1.35)	n = 72	0.81 (0.39-1.66)	0.92 (0.43-1.93)
Endothelial score	Low	n = 86	0.61 (0.33-1.15)	n = 78	0.67 (0.34-1.31)	0.71 (0.35-1.46)
	Inter.	n = 84	**0.21 (0.09-0.51)**	n = 76	**0.19 (0.07-0.49)**	**0.22 (0.09-0.58)**
	High	n = 81	0.58 (0.27-1.25)	n = 66	0.57 (0.25-1.32)	0.55 (0.23-1.31)
Fibroblasts score	Low	n = 78	**0.50 (0.27-0.93)**	n = 71	0.56 (0.29-1.07)	0.62 (0.32-1.20)
	Inter.	n = 86	**0.36 (0.17-0.77)**	n = 75	**0.30 (0.13-0.71)**	**0.30 (0.13-0.72)**
	High	n = 87	0.53 (0.22-1.29)	n = 74	0.51 (0.20-1.28)	0.42 (0.16-1.13)

Multivariable Cox regression analysis of RFS across cell abundance scores. aHRs compare tamoxifen treatment vs. no endocrine therapy across three models: Full cohort, Reduced: Excludes patients with missing/uncertain ER status (n=58), Adjusted: Reduced cohort with additional adjustment for ER percentage (ER%). TME, Tumour Microenvironment; RFS, Recurrence-Free Survival; aHR, Adjusted Hazard Ratio; CI, Confidence Interval; ER, Estrogen Receptor.
