# Supplementary material for: The tumour microenvironment influences long-term tamoxifen benefit in postmenopausal ER+/HER2− breast cancer patients: a secondary analysis of the randomised Stockholm Tamoxifen (STO-3) trial
Source: BMC Med. 2026 Aug 5;24:424. doi: 10.1186/s12916-026-05091-5 (PMC13445795; doi:10.1186/s12916-026-05091-5)
Supplement: Supplementary file 5 [file 12916_2026_5091_MOESM5_ESM.pdf]

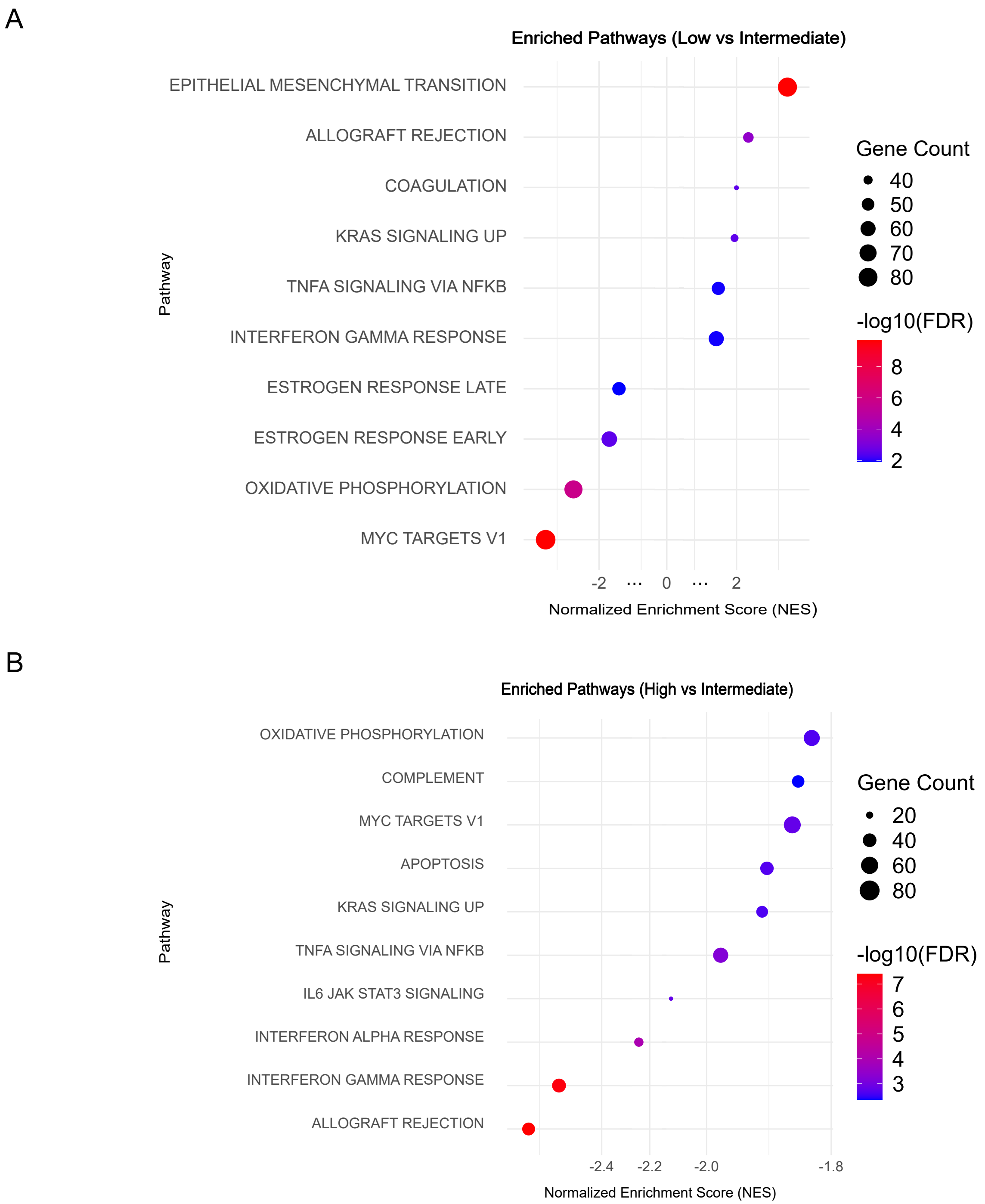
A
Enriched Pathways (Low vs Intermediate)
EPITHELIAL MESENCHYMAL TRANSITION
ALLOGRAFT REJECTION
COAGULATION
KRAS SIGNALING UP
TNFA SIGNALING VIA NFKB
INTERFERON GAMMA RESPONSE
ESTROGEN RESPONSE LATE
ESTROGEN RESPONSE EARLY
OXIDATIVE PHOSPHORYLATION
MYC TARGETS V1
Pathway
-2 ⋯ 0 ⋯ 2
Normalized Enrichment Score (NES)
Gene Count
40
50
60
70
80
-log10(FDR)
8
6
4
2
B
Enriched Pathways (High vs Intermediate)
OXIDATIVE PHOSPHORYLATION
COMPLEMENT
MYC TARGETS V1
APOPTOSIS
KRAS SIGNALING UP
TNFA SIGNALING VIA NFKB
IL6 JAK STAT3 SIGNALING
INTERFERON ALPHA RESPONSE
INTERFERON GAMMA RESPONSE
ALLOGRAFT REJECTION
Pathway
-2.4
-2.2
-2.0
-1.8
Normalized Enrichment Score (NES)
Gene Count
20
40
60
80
-log10(FDR)
7
6
5
4
3
